# Quality of life profile of general Vietnamese population using EQ-5D-5L

**DOI:** 10.1186/s12955-017-0771-0

**Published:** 2017-10-11

**Authors:** Long Hoang Nguyen, Bach Xuan Tran, Quynh Ngoc Hoang Le, Tung Thanh Tran, Carl A. Latkin

**Affiliations:** 1 School of Medicine and Pharmacy, Vietnam National University, Hanoi, Vietnam; 2 Institute for Preventive Medicine and Public Health, Hanoi Medical University, Hanoi, Vietnam; 3 Johns Hopkins Bloomberg School of Public Health, United States of America, Baltimore, MD USA; 4 Faculty of Pharmacy, Duy Tan University, Da Nang, Vietnam; 5 Institute for Global Health Innovations, Duy Tan University, Da Nang, Vietnam

**Keywords:** EQ-5D-5L, Health-related quality of life, Population, Norms, Vietnam

## Abstract

**Background:**

Health-related quality of life (HRQOL) is a vital benchmark to assess the effects of health interventions and policies. Measuring HRQOL of the general population is essential to establish a reference for health outcomes evaluations. However, evidence on HRQOL of general populations in low and middle income countries is very limited. This study aimed to measure HRQOL of the Vietnamese population by using the EuroQol-5 dimensions-5 levels (EQ-5D-5L) instrument and determine its associated factors.

**Methods:**

A cross-sectional study was performed in Hanoi with 1571 residences in Hanoi, the capital city of Vietnam. EQ-5D-5L and EQ- visual analogue scale (EQ-VAS) were used to assess HRQOL. Potential covariates included socio-demographic characteristics, having acute symptoms in the last four weeks, chronic diseases in the last three months, having multiple health issues, and health service utilisation in the last twelve months. A generalized linear model was employed to identify the association between HRQOL and covariates.

**Results:**

Overall, the mean EQ-5D utility index was 0.91 (SD = 0.15), and the mean EQ-VAS score was 87.4 (SD = 14.3). The highest proportion of respondents reporting any problems was in Usual activities (24.3%), followed by Anxiety/Depression (15.2%) and Pain/Discomfort (10.0%), while the lowest percentage was in Self-care (2.5%). Lower HRQOL composite scores were related to unemployment, lower income, higher education, living in urban areas, having chronic diseases, having multiple health issues and using health service. For any health problem self-reported by respondents, the health utility reduced by 0.02 (respiratory diseases) to 0.15 (musculoskeletal diseases).

**Conclusions:**

Health utility of the general population and reductions for self-reported health problems in this study are useful for future population health evaluations and comparisons. It also informs the development of interventions to reduce health problems of the general population.

## Background

Self-reported health-related quality of life (HRQOL) is commonly used for monitoring the health status of the general population and inform the effectiveness of treatments or health care policies [1, 2]. Evaluating the HRQOL of the general population can enable to compare the health status of the general population and specific patient groups to estimate the burden of different diseases as regards HRQOL [3]. In economic evaluations, HRQOL of the general population can play a role as a reference group to assess the incremental effectiveness of interventions if the control groups do not exist [4]. Moreover, in the realm of policy development, these HRQOL data can be used to support policy makers identifying policy gaps and inequalities for fulfilling, and detecting priorities to allocate resources [5, 6]. Therefore, assessing HRQOL of the general population to construct population norms is becoming a necessity for the development of healthcare in each country.

Nonetheless, HRQOL itself does not reflect the clinically important differences in the treatments or policies directly. A term “minimal clinical important difference” (MCID) has been raised to provide such information. Beyond statistical significance, this term refers to the change of outcome that is large enough to be beneficial for the patients; and is worthy for patients to repeat the intervention or treatment if they have an opportunity to take again [7–9]. Methods to measure MCID can be classified into two groups: anchor-based and distribution-based [10]. The distribution-based used statistical features of the sample namely 0.5 standard deviation (0.5SD), effect size and one standard error of measurement (1SEM) as thresholds to detect clinical differences [10]. Meanwhile, anchor-based methods employ external indicators such as self-reported health outcomes and biological measurements [11]. Of which, health-related quality of life (HRQOL) has been broadly used to evaluate the MCID because it reflects the perceptions of patients about their physical and mental health and what the values of HRQOL are meaningful for them [8].

Previously, a general preference-based measure so-called EuroQol- 5 Dimensions – 3 Levels (EQ-5D-3L) was widely used to estimate health utility and HRQOL [4]. This tool describes the health status of respondents in five dimensions: mobility, self-care, usual activities, pain/discomfort and anxiety/depression, with three options to respond: no problem, moderate problems and severe problems [12]. However, its high ceiling effect becomes a major drawback that limits capacities to capture clinical differences regarding HRQOL [13]. A new instrument entitled EQ-5D-5 L, with five levels of response, is then introduced to replace EQ-5D-3 L. It has been proven that can reduce the ceiling effect and have high convergent validity, more sensitivity and is feasible to use in both clinical and community settings [14–19].

Due to the social and cultural differences, EQ-5D-5 L population norms have been reported and validated in many countries such as Spain [20], Australia [21], UK [16], Germany [17], Uruguay [14], Poland [22], Canada [23], and Japan [18]. Younger people, males, higher income, higher education, employed and married people were more likely to have better HRQOL [14, 16, 18, 21, 22, 24, 25]. In Vietnam, EQ-5D-5 L has been employed to measure HRQOL among specific populations such as HIV positive patients [6, 19], patients receiving methadone therapy [26–30], Vietnamese youths [31–33] and residences in mountainous settings [34]. However, there is none of the evidence on the HRQOL of general Vietnamese population. Therefore, this study aimed to profile the health status of general Vietnamese people living in Hanoi by using the EQ-5D-5 L instrument, and identify its associated factors.

## Methods

### Study designs and participants

A cross-sectional study was conducted in October 2015 in Hanoi, a capital of Vietnam. Hanoi is the biggest city of Vietnam with covering more than 3300 km^2^ and approximately 7.7 million people living in 30 districts and 584 communes until April 2017. The density of population is 2300 people/km^2^ [35].

In this study, we selected randomly 176 communes in 29/30 districts as study settings. In each commune, we randomly selected ten people who had met following criteria: 1) Aged from 15 years old or above; 2) Agreeing to enroll in this study, and 3) Having ability to answer the survey. We approached eligible subjects, introduced about the research and invited them to participate in the study. People who accepted to enroll were asked to give written informed consent. A total of 1760 residents participated in the study; however, after excluding people who did not answer the EQ-5D-5 L instrument completely, the remaining sample size of this study was 1571 (89.3%). No difference was found between included and excluded respondents in accordance with socio-economic characteristics.

### Measures and instruments

Face-to-face interviews were conducted by well-trained undergraduate and post-graduate students in the field of Public Health. We developed a structured questionnaire to collect data from respondents. The variables of interest were described as below:

#### EQ-5D-5L

In this study, HRQOL of participants was measured by using EuroQol-5 dimensions-5 levels (EQ-5D-5L). Five dimensions of this tool include Mobility, Self-care, Usual Activities, Pain/Discomfort and Anxiety/Depression, which have five levels of response: from no problems (code 1) to extreme problems (code 5). These levels of each dimension can be combined to identify 3125 possible health states from 11111 (full health) to 55555 (worst health) [36]. Each health state defines one single “utility” score, which can be transformed by using the interim scoring for EQ-5D-5 L. In the current study, due to the unavailability of Vietnamese cross-walk value set, we used the Thailand value set with the score ranged from −0.451 to 1 [36]. Moreover, another part of EQ-5D-5 L is a visual analogue scale (EQ-VAS), which can be used assesses the self-rated health of respondents by using a 100-mm scale with the score ranged from 0 (the worst health you can imagine) to 100 (the best health you can imagine). The Vietnamese version of EQ-5D-5 L has been used and validated in elsewhere [19].

#### Other characteristics

In this study, we collected socio-demographic characteristics of interest included age, living area, educational attainment, employment status, gender, total household income; and marital status. The household income was then separated into five quintiles from poorest to richest. We also asked participants to report whether they suffered acute symptoms in the last 4 weeks, having chronic diseases in the last 3 months and used health services in the last 12 months.

#### Statistical analysis

Data analysis was performed using Stata version 12.0 (Stata Corp. LP, College Station, United States of America). We described the socio-economic status, EQ-5D-5 L profiles, utility score and VAS scores according to age groups and gender. Due to the non-normal distribution of utility and VAS scores (Kolmogorov-Smirnov test, *p* < 0.05), the differences of utility and VAS scores between different groups were tested by employing Mann-Whitney (for gender, living location, having acute symptoms in the past 4 weeks, having chronic diseases in the past 3 months and using health service in the past 12 months), and Kruska-wallis tests (for age groups, marital status, education, occupation, income quintiles, and number of health issues). Mann-whitney test was also used to test the differences of EQ-5D-5 L index and EQ-VAS score according to different dimensions of the EQ-5D-5 L instrument. Spearman’s correlation coefficient was also conducted to identify the relationship between utility score and VAS score. Correlations were classified in three categories: weak (rh0 < 0.3); moderate (0.3 < rh0 < 0.5); and strong (>0.5) [37]. *P*-value <0.05 was considered statistical significance.

We detected the between-group MCID by using ANOVA test to compare the HRQOL of respondents with and without specific health conditions/diseases. The anchor-based approach has been argued that can be used in both cross-sectional and longitudinal designs [18, 38]. In this study, we only included health conditions for which more than 10 participants had responded positively. This approach has been used in the previous study in Japan [18], which might assure to reliably detect the minimal differences of HRQOL between people with and without health conditions. However, we found that only the number of patients with hypertension met this criterion. Therefore, we decided to group the diseases into four categories: hypertension, respiratory diseases, musculoskeletal diseases and gastrointestinal diseases. Other categories such as cardiovascular diseases, endocrinology diseases, etc. did not have enough 10 respondents; thus, we did not include in the analysis.

Generalized linear model (GLM), which could manage skewness and heteroscedasticity, was employed to explore relationships between potential covariates and EQ-5D utility scores as well as VAS scores [39, 40]. Due to the requirement of the model, because EQ-5D utility score may contain negative numbers, we computed the *EQ-5D-5L disutility score* (1-utility score) and used this score as a dependent variable in the model (model 1) [41]. Therefore, if the coefficient of one factor in the model is positive, it means that this factor can increase the disutility score; or decrease the utility score of EQ-5D-5 L. We also divided EQ-VAS score to 100 and used the new variable as a dependent variable (model 2). Potential explanatory variables included age, living area, educational attainment, employment status, gender, household income quintiles (poorest to richest), marital status, having acute symptoms in the last 4 weeks, having chronic diseases in the last 3 months, number of health issues, and using health service in the last 12 months.

GLM models required accompanying distribution family and link function. Modified Park tests were used to determine data’s distribution family based on the lowest χ^2^ values [41]. Three distribution families were tested including Gamma, Gaussian and Poisson, of which the Poisson family was the most appropriate family to describe the EQ-5D-5 L disutility and EQ-VAS score’s distributions. In addition, we also identied the fitted link function for GLM models among three types (identity, squareroot and log links). By using Peason correlation test, Pregibon link test and Hosmer-Lemeshow test [40, 42], we found that the log was the most proper link function for the models. Theoretically, the log link function exponentiates the combination of predictors instead of log transforming the outcome data [41].

Reduced models were developed by using stepwise forward selection strategies, that variables were included based on the *p*-value <0.1 of the log-likelihood ratio tests [43].

## Results

A total of 1571 individuals enrolled into the study. Most of the participants were female (61.5%), adults aged from 25 to 44 (53.1%), living with spouse/partner (65.5%) and living in the urban area (86.0%). The majority of respondents were at or having an undergraduate education (52.5%). White-collars were predominant jobs with 31.3%, followed by the Blue-collars (17.5%) and students (16.7%). Table 1 also shows that only 2.3%, 6.3% and 8.3% respondents had acute symptoms in the last 4 weeks, chronic diseases in the last 3 months and had health issues, respectively. About one-fifth of participants utilized health care services in the last 12 months.

**Table 1:** Demographic characteristics of respondents

Characteristics	Female		Male		Total	
	n	%	n	%	n	%
Total	964	61.5	603	38.5	1567	100.0
Age groups						
15–24	195	20.2	135	22.4	330	21.1
25–34	317	32.9	202	33.5	519	33.1
35–44	195	20.2	119	19.7	314	20.0
45–54	139	14.4	76	12.6	215	13.7
55–64	78	8.1	51	8.5	129	8.2
65+	40	4.2	20	3.3	60	3.8
Marital status						
Single	266	27.7	244	40.5	510	32.6
Live with spouse/partner	677	70.4	347	57.6	1024	65.5
Divorce/Separate/Widow	19	2.0	11	1.8	30	1.9
Education						
Secondary school or less	125	13.0	78	13.0	203	13.0
High school	301	31.3	194	32.4	495	31.7
Undergraduate	517	53.7	302	50.5	819	52.5
Postgraduate	19	2.0	24	4.0	43	2.8
Occupation						
Student	161	16.7	100	16.6	261	16.7
Blue-collar	127	13.2	147	24.4	274	17.5
White-collar	315	32.7	175	29.1	490	31.3
Retired	64	6.7	50	8.3	114	7.3
Housework	197	20.5	13	2.2	210	13.4
Unemployed	13	1.4	19	3.2	32	2.0
Others (freelancers, farmers, etc.)	86	8.9	98	16.3	184	11.8
Living locations						
Rural	121	12.6	98	16.3	219	14.0
Urban	843	87.5	505	83.8	1348	86.0
Having acute symptoms in the past 4 weeks						
Yes	28	2.9	8	1.3	36	2.3
No	936	97.1	592	98.7	1528	97.7
Having chronic diseases in the past 3 months						
Yes	63	6.5	36	6.0	99	6.3
No	901	93.5	564	94.0	1465	93.7
Number of health issues						
0	878	91.1	558	92.7	1437	91.7
1	78	8.1	42	7.0	120	7.7
2	8	0.8	2	0.3	10	0.6
Using health services in the past 12 months						
Yes	173	18.0	131	21.7	304	19.4
No	791	82.1	472	78.3	1263	80.6

**Fig. 1:**
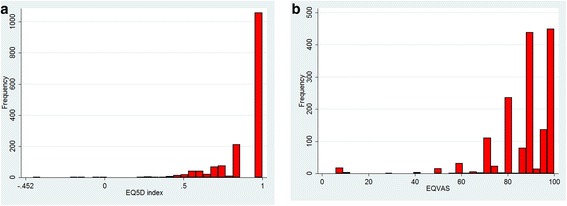
Distribution of EQ5D index and EQVAS. **a** EQ5D index; **b** EQVAS

Fig. 1a illustrates that EQ-5D index was ranged from −0.452 to 1, which was left-skewed with the dominant value at 1.00 (i.e. “Full health”). Similarly, Fig. 1b reveals that EQ-VAS score was ranged from 6 to 100, which was also left-skewed with the major clustering from 80 to 100 (i.e. “the best health you can imagine”).

Table 2 depicts profiles of EQ-5D-5 L domains according to frequencies of each item response. The highest proportion of respondents reporting any problems was in Usual Activities (24.3%), followed by Anxiety/Depression (15.2%) and Pain/Discomfort (10.0%), while the lowest percentage was in Self-care (2.5%).

**Table 2:** Profiles of EQ-5D-5 L by age group

Domains	15–24		25–34		35–44		45–54		55–64		65+		Total	
	n	%	n	%	n	%	n	%	n	%	n	%	n	%
Mobility														
No problems	317	96.1	501	96.2	302	95.9	198	92.1	118	91.5	50	82.0	1486	94.6
Slight problems	10	3.0	14	2.7	13	4.1	14	6.5	9	7.0	9	14.8	69	4.4
Moderate problems	3	0.9	3	0.6	0	0.0	2	0.9	2	1.6	1	1.6	11	0.7
Severe problems	0	0.0	3	0.6	0	0.0	0	0.0	0	0.0	0	0.0	3	0.2
Unable to walk about	0	0.0	0	0.0	0	0.0	1	0.5	0	0.0	1	1.6	2	0.1
Self-care														
No problems	323	97.9	514	98.7	309	98.1	208	96.7	123	95.4	54	88.5	1531	97.5
Slight problems	5	1.5	4	0.8	5	1.6	4	1.9	6	4.7	6	9.8	30	1.9
Moderate problems	0	0.0	1	0.2	1	0.3	2	0.9	0	0.0	0	0.0	4	0.3
Severe problems	1	0.3	2	0.4	0	0.0	1	0.5	0	0.0	0	0.0	4	0.3
Extreme problems	1	0.3	0	0.0	0	0.0	0	0.0	0	0.0	1	1.6	2	0.1
Usual activities														
No problems	259	78.5	409	78.5	236	74.9	164	76.3	87	67.4	34	55.7	1189	75.7
Slight problems	67	20.3	108	20.7	79	25.1	49	22.8	40	31.0	23	37.7	366	23.3
Moderate problems	2	0.6	3	0.6	0	0.0	2	0.9	2	1.6	3	4.9	12	0.8
Severe problems	0	0.0	0	0.0	0	0.0	0	0.0	0	0.0	0	0.0	0	0.0
Unable to do	2	0.6	1	0.2	0	0.0	0	0.0	0	0.0	1	1.6	4	0.3
Pain/Discomfort														
No pain	308	93.3	481	92.3	277	87.9	193	89.8	104	80.6	51	83.6	1414	90.0
Slight pain	19	5.8	37	7.1	34	10.8	14	6.5	24	18.6	6	9.8	134	8.5
Moderate pain	1	0.3	2	0.4	4	1.3	6	2.8	1	0.8	2	3.3	16	1.0
Severe pain	2	0.6	1	0.2	0	0.0	0	0.0	0	0.0	0	0.0	3	0.2
Extreme pain	0	0.0	0	0.0	0	0.0	2	0.9	0	0.0	2	3.3	4	0.3
Anxiety/Depression														
Not anxious/depressed	284	86.1	455	87.3	254	80.6	183	85.1	107	83.0	49	80.3	1332	84.8
Slightly	34	10.3	59	11.3	54	17.1	25	11.6	20	15.5	8	13.1	200	12.7
Moderately	10	3.0	5	1.0	5	1.6	4	1.9	2	1.6	1	1.6	27	1.7
Severely	1	0.3	1	0.2	1	0.3	1	0.5	0	0.0	2	3.3	6	0.4
Extremely	1	0.3	1	0.2	1	0.3	2	0.9	0	0.0	1	1.6	6	0.4

The mean EQ-5D utility scores according to different characteristics of respondents were summarized in Table 3. Overall, the mean EQ-5D utility index was 0.91 (SD = 0.15). Lower utility scores were observed in females, higher age groups, and lower income quintiles (*p* < 0.01). Participants who were divorced/separated/widow had the lowest utility score (mean = 0.84, SD = 0.20) compared to other marital categories (*p* < 0.01). Additionally, people who were retired had a lower utility score (mean = 0.85, SD = 0.16) in comparison to other people (*p* < 0.01). Similarly, people suffering chronic diseases or had any health issues had significantly lower utility scores than their counterparts (*p* < 0.05). Meanwhile, no statistically significant difference in utility scores was found regarding educational attainment, living location, having acute symptoms in the past 4 weeks and using health service in the past 12 months.

**Table 3:** EQ-5D-5L utility scores by different characteristics

	Total			Female		Male	
	Mean	SD	*p*-value	Mean	SD	Mean	SD
Total	0.91	0.15		0.91^a^	0.14	0.92	0.15
Age groups							
15–24	0.92	0.14	<0.01	0.92	0.14	0.92	0.15
25–34	0.92	0.13		0.92	0.14	0.94	0.12
35–44	0.91	0.14		0.90	0.14	0.91	0.14
45–54	0.90	0.17		0.90	0.16	0.90	0.20
55–64	0.88	0.16		0.87	0.16	0.89	0.15
65+	0.81	0.25		0.83	0.16	0.84	0.24
Marital status							
Single	0.92	0.15	<0.01	0.92	0.14	0.93	0.15
Live with spouse/partner	0.91	0.14		0.90	0.14	0.91	0.14
Divorce/Separate/Widow	0.84	0.20		0.86	0.18	0.80	0.23
Education							
Secondary school or less	0.90	0.16	0.91	0.89	0.16	0.92	0.16
High school	0.91	0.14		0.90	0.15	0.93	0.14
Undergraduate	0.91	0.14		0.91	0.14	0.91	0.15
Postgraduate	0.90	0.15		0.89	0.16	0.90	0.14
Occupation							
Student	0.90	0.15	<0.01	0.90	0.16	0.90	0.16
Blue-collar	0.93	0.14		0.91	0.14	0.94	0.13
White-collar	0.92	0.14		0.92	0.13	0.93	0.15
Retired	0.85	0.16		0.86	0.16	0.85	0.17
Housework	0.88	0.16		0.88	0.16	0.84	0.18
Unemployed	0.89	0.18		0.88	0.18	0.90	0.18
Others (freelancers, farmers, etc.)	0.94	0.12		0.94	0.11	0.94	0.14
Income quintiles							
Poorest	0.88	0.17	<0.01	0.88	0.17	0.89	0.16
Poor	0.90	0.15		0.89	0.14	0.91	0.17
Middle	0.92	0.14		0.91	0.14	0.92	0.13
Rich	0.93	0.11		0.94	0.10	0.93	0.12
Richest	0.94	0.15		0.94	0.10	0.94	0.17
Living locations							
Rural	0.91	0.13	0.89	0.89	0.15	0.94	0.11
Urban	0.91	0.15		0.91	0.14	0.91	0.16
Having acute symptoms in the past 4 weeks							
Yes	0.87	0.17	0.09	0.85	0.18	0.94	0.10
No	0.91	0.15		0.91	0.14	0.92	0.15
Having chronic diseases in the past 3 months							
Yes	0.83	0.19	<0.01	0.82	0.18	0.83	0.20
No	0.91	0.15		0.91	0.14	0.92	0.14
Number of health issues							
0	0.92	0.15	<0.01	0.91	0.14	0.92	0.14
1	0.85	0.18		0.85	0.17	0.84	0.19
2	0.78	0.25		0.72	0.25	1.00^b^	0.00
Using health services in the past 12 months							
Yes	0.91	0.15	0.07	0.91	0.14	0.92	0.14
No	0.89	0.16		0.89	0.16	0.91	0.16

^a^ *p*-value = 0.03 (Mann-Whitney test): compare the difference of utility score between male and female

^b^ There are only two males in this category

Table 4 provides summaries of the EQ-VAS score according to different socio-demographic characteristics. The mean EQ-VAS score was 87.4 (SD = 14.3) with the statistically higher score in male compared to female. We also found significant differences on EQ-VAS scores in term of age groups, marital status, education attainment, occupation, living location, having acute and chronic diseases, having health issues, and using health care services (*p* < 0.01).

**Table 4:** EQ-VAS scores by different characteristics

	Total			Female (*n* = 964)		Male (*n* = 603)	
	Mean	SD	*p*-value	Mean	SD	Mean	SD
Total	87.4	14.3		87.1^a^	14.2	88.0	14.6
Age groups							
15–24	88.5	15.8	<0.01	87.1	18.0	90.5	11.6
25–34	88.1	14.5		88.6	12.3	87.3	17.3
35–44	87.5	12.4		87.2	12.6	88.1	12.1
45–54	85.8	15.0		85.3	14.5	86.9	15.8
55–64	86.4	13.1		85.8	13.5	87.4	12.6
65+	83.3	13.7		82.6	12.3	83.9	16.3
Marital status							
Single	88.2	16.3	<0.01	87.6	16.9	88.9	15.7
Live with spouse/partner	87.2	13.1		86.9	12.9	87.7	13.4
Divorce/Separate/Widow	82.3	18.5		85.3	16.2	77.3	21.8
Education							
Secondary school or less	86.3	12.3	<0.01	85.1	12.2	88.2	12.3
High school	87.2	13.4		87.3	12.3	87.2	15.1
Undergraduate	88.1	14.9		87.5	15.5	89.2	13.9
Postgraduate	83.5	20.1		85.8	14.5	81.7	23.8
Occupation							
Student	88.0	16.0	<0.01	86.4	18.4	90.6	10.6
Blue-collar	88.0	12.9		87.4	12.4	88.6	13.4
White-collar	89.2	14.0		88.7	13.4	90.1	15.0
Retired	83.5	12.5		84.5	11.4	82.7	13.8
Housework	86.1	13.4		86.5	12.4	79.7	23.4
Unemployed	84.1	15.4		86.2	11.6	82.7	17.7
Others (freelancers, farmers, etc.)	85.6	16.0		85.4	15.7	85.7	16.3
Income quintiles							
Poorest	86.4	13.3	0.24	86.2	13.6	86.9	12.9
Poor	86.2	16.7		86.1	17.1	86.6	16.1
Middle	88.7	10.8		88.2	11.4	89.8	9.3
Rich	87.2	15.8		88.2	10.3	85.9	21.0
Richest	86.9	17.7		86.6	17.7	87.1	17.7
Living locations							
Rural	90.2	10.0	<0.01	88.5	10.9	92.2	8.4
Urban	87.0	14.9		86.9	14.6	87.2	15.4
Having acute symptoms in the last 4 weeks							
Yes	75.8	17.9	<0.01	73.1	18.4	85.5	12.2
No	87.7	14.1		87.5	13.8	88.0	14.7
Having chronic diseases in the last 3 months							
Yes	78.1	18.5	<0.01	79.8	15.1	75.0	23.1
No	88.0	13.8		87.6	14.0	88.8	13.6
Number of health issues							
0	88.3	13.6	<0.01	87.9	13.7	88.9	13.6
1	78.5	18.0		79.4	15.6	76.7	21.9
2	70.5	17.1		69.4	18.2	75.0	21.2
Using health services in the last 12 months							
Yes	82.3	19.2	<0.01	82.1	18.7	82.5	20.0
No	88.7	12.6		88.2	12.7	89.6	12.3

^a^ *p*-value = 0.04 (Mann-Whitney test): compare the difference of EQ-VAS score between male and female

Table 5 shows that the EQ-VAS and EQ-5D-5 L utility scores were varied significantly according to whether the respondents reported any problems or not in each dimension. Overall, regarding EQ-VAS, people having problem-free had 6 points higher than those who reported at least one problem; meanwhile, regarding EQ-5D-5 L utility score, respondents reporting at least one problem had 0.28 point lower than those not having any problems (*p* < 0.05).

**Table 5:** EQ-5D-5L utility score and EQ-VAS score by different domains of EQ-5D-5 L instrument

Domain	EQ-VAS score			EQ-5D-5L utility score		
	Mean	SD	*p*-value	Mean	SD	*p*-value
Mobility						
No problem	88.2	13.8	<0.01	0.93	0.11	<0.01
Having problems	74.3	16.7		0.53	0.19	
Self-care						
No problem	87.8	14.0	<0.01	0.92	0.12	<0.01
Having problems	71.5	19.3		0.41	0.22	
Usual activities						
No problem	88.3	14.3	<0.01	0.97	0.10	<0.01
Having problems	84.5	14.0		0.71	0.14	
Pain/discomfort						
No problem	88.6	13.8	<0.01	0.94	0.10	<0.01
Having problems	76.6	14.9		0.61	0.17	
Anxiety/Depression						
No problem	88.8	13.9	<0.01	0.95	0.10	<0.01
Having problems	79.6	14.5		0.67	0.16	
All domains						
Problem-free	89.3	14.2	<0.01	1.00	0.00	<0.01
At least one problem	83.5	13.8		0.72	0.13	

Table 6 shows the between-group MCID in our sample. Regarding EQ-5D utility score, the significant MID (*p* < 0.05) were found between respondents with and without hypertension, musculoskeletal diseases, having chronic diseases in the last 3 months and having multiple health issues. Meanwhile, for EQ-VAS score, the significant MCID were found between respondents with and without respiratory diseases, musculoskeletal diseases, having acute symptoms in the last 4 weeks, having chronic diseases in the last 3 months and having multiple health issues.

**Table 6:** Between-group MCID of EQ-VAS and EQ-5D utility scores for different health conditions

Health issue	n	EQ-VAS score		EQ-5D utility score	
		Diff^a^	95% CI	Diff^a^	95% CI
Intercept	1567	84.7		0.91	
Common diseases					
No diseases	1437	–	–	–	–
Hypertension	18	−3.6	−10.3; 3.04	−0.12*	−0.19; − 0.05
Respiratory diseases	12	−18.6*	−26.7; 019.5	−0.02	−0.11; 0.07
Musculoskeletal diseases	31	−11.1*	−16.2; −6.0	−0.15*	−0.20; −0.09
Gastrointestinal diseases	23	−5.3	−11.2; 0.6	−0.04	−0.10; 0.02
Having acute symptoms in the last 4 weeks	36	−11.8*	−16.6; −7.1	−0.04	−0.09; 0.01
Having chronic diseases in the last 3 months	99	−10.0*	−12.8; −7.1	−0.09*	−0.12; −0.06
Number of health issues					
0	1437	–	–	–	–
1	120	−9.8*	−13.0; −6.7	−0.07*	−0.10; −0.04
2	10	−17.8*	−28.2; −7.3	−0.14*	−0.25; −0.03

^a^Difference between respondents with and without health conditions/diseases

* *p*< 0.05

Table 7 shows ten most common EQ-5D-5 L health states, which accounted for 93.8% of respondents. Health states “11111” (full health), “11112” (slightly problems in anxiety/depression) and “12111” (slightly problems in self-care) were the most frequent responses in the sample. However, we only found a low and positive correlation between utility scores and EQ-VAS scores (rh0 = 0.2850, *p* < 0.01).

**Table 7:** Most frequent EQ-5D-5 L health states with mean utility scores and EQVAS scores

Health states	Number	Percent	Cum%	Mean Utility	Mean VAS
11111	1058	67.4	67.4	1.00	89.3
11112	211	13.4	80.8	0.79	89.6
12111	54	3.4	84.2	0.81	81.4
12112	45	2.9	87.1	0.73	86.1
22111	34	2.2	89.2	0.72	83.6
22112	24	1.5	90.8	0.67	82.2
21112	15	1.0	91.7	0.70	81.7
21111	11	0.7	92.4	0.78	82.7
22222	11	0.7	93.1	0.47	75.0
11212	10	0.6	93.8	0.66	68.5

In the reduced multivariate generalized linear models, the EQ-5D-5 L disutility scores were found higher among those retired or having housework, having higher education, being at lower income quintiles and having chronic diseases in the last 3 months. Meanwhile, living in urban area, having chronic diseases, having a higher number of health issues, and utilizing health care services were negatively associated with the EQ-VAS score. Nevertheless, people who were at middle-income quintile, having full/part-time jobs or being a student were found to have better health status regarding EQ-VAS (Table 8).

**Table 8:** Generalized linear models of EQ-5D-5 L disutility scores, EQ-VAS scores, and different characteristics

	EQ-5D-5L disutility score		EQ-VAS scores	
	Coef.	95% CI	Coef.	95% CI
Age groups (vs 15–25)				
65+	0.39*	−0.05; 0.84		
Occupation (vs Unemployed)				
Full/part time, student			0.17**	0.00; 0.33
Retired	0.40***	0.08; 0.73		
Housework	0.51***	0.25; 0.78		
Education (vs Secondary school or less)				
≥ Undergraduate	0.27***	0.07; 047		
Income quintiles (vs Poorest)				
Middle	−0.24**	−0.45; −0.03	0.22***	0.08; 0.36
Rich	−0.50***	−0.83; −0.18		
Richest	−0.52***	−0.88; −0.16		
Living locations (Urban vs Rural)			−0.35***	−0.54; −0.15
Having acute symptoms in the last four weeks (Yes vs No)	0.38*	−0.07; 0.83		
Having chronic diseases in the last 3 months (Yes vs No)	0.69***	0.39; 0.98	−0.49***	−0.77; −0.21
Number of health issues (vs No issue)				
1			−0.54***	−0.80; −0.27
2			−0.75**	−1.34; −0.16
Using health services in the last 12 months (Yes vs No)			−0.38***	−0.59; −0.16
Constant	−2.49***	−2.70; −2.29	2.21***	1.99; 2.44
AIC	0.55		1.91	
BIC	−9520		−9780	

*p < 0.1; **p < 0.05; ***p < 0.01

## Discussion

In our knowledge, this is the first study that offers critical insights into HRQOL of Vietnamese people, informing evidence for monitoring changes in health strategies and evaluating the effectiveness of public health intervention in the future. Generally, the mean EQ-5D utility score in our sample was 0.91 (SD = 0.15), which was consistent with the utility scores of different populations worldwide measured by EQ-5D-5 L such as populations in Australian (0.90) [21]; German (0.92) [17]; Italian (0.92) [44], but lower than that of Uruguayan (0.95) [14] and Polish (0.96) [22]. The differences may be explained due to the difference of cross-walk value set used as well as cultural and social distinctions [15, 45, 46]. Notably, using the cross-walk value from Thailand instead of Vietnam is a major disadvantage of this study; however, the value set of Vietnamese preference is not available currently. Guidelines emphasized the need of having a specific value set for each country due to the cultural differences [21, 36]. Therefore, in order to estimate HRQOL of Vietnamese people precisely, a direct measure of the value set for Vietnamese people is recommended and should be warranted in further studies.

In this study, we found that our sample had greater problems in usual activities and anxiety/depression (24.3% and 15.2%, respectively). This result was similar to a previous study in Vietnam, which used EQ-5D-3 L and showed that the percentage of people having anxiety/ depression was the highest [3]. In Australia, Germany, and Spain, the major problems were pain/ discomfort and mobility [20, 21], while in Poland, most of the respondents reported problems in pain/ discomfort and anxiety/depression. Noticeably, most of our sample (67.4%) reported the perfect health state (11111). Although this figure indicates the benefit of EQ-5D-5 L in reducing ceiling effect compared to the previous data using EQ-5D-3 L (with >85% reporting no problems in all dimensions) [3], the ceiling effect of this instrument remained strong. This result in our sample was similar to the result in Spanish population with 62.5% [20], but it is much higher than in other countries such as Australia (42.8%), UK (47.6%), Germany (47.5%), Uruguay (44.0%), and Poland (38.5%) [14, 17, 21, 22].

Meanwhile, only 22.8% people reported their VAS score at 100 points (“the best health state that you can imagine”), suggesting that the EQ-VAS is more proper than EQ-5D-5 L in measuring global health rating [16, 47]. Indeed, EQ-VAS has been used widely in monitoring the self-rated health of patients and populations over time along with biomedical and behavioral indicators [19, 28, 30, 47, 48]. EQ-VAS has good psychometric properties and is a simple tool to use. Further, this tool does not depend on any value sets [36]. However, EQ-VAS is less recommended to use in health economic evaluations than other direct preference-based measure namely time trade-off and standard gamble [49, 50]. Tran et al. in their systematic review argued that EQ-VAS might only reflect the alterations of perceptions of respondents rather than their real health status [51]. The authors suggested that EQ-VAS should be incorporated into other indirect preference-based measure such as EQ-5D-5 L to determine the short-term and long-term change of HRQOL more accurately [51]. As the correlation between EQ-5D utility score and the EQ-VAS score was low in this study, we confirmed the need for combining EQ-VAS and EQ-5D-5 L when conducting research in Vietnamese populations.

In the current study, we used the anchor-based approach by using the EQ-5D-5 L and EQ-VAS instruments to measure the between-group MCID between participants with and without specific health conditions/diseases. Generally, participants with any diseases, symptoms, and those people with increasing health issues had lower HRQOL. These findings were similar to the results from previous population-based surveys in Canada, Germany, and Japan [18, 52, 53]. The MCID of the EQ-5D-5 L was 0.07 in people with one health issue and 0.14 in respondents with two health issues. These are consistent with the study in Canada, which indicated a decrease of 0.07 in people having one morbidity and 0.11 among those having two morbidities [52]. Notably, the results may be different from the intra-respondent MCID because the anchor-based method is commonly used in longitudinal studies, which measure the outcomes in multiple points of time. However, we aware that repeating surveys with a large sample size of the general population is burdensome. Moreover, the between-group MCID could provide useful information with the appropriate intepretation [18]. Nonetheless, further understand about the MCID of each disease should be warranted in order to achieve optimal disease management.

Despite the significant differences in univariate analysis, multivariate models show that age, gender, and marital status were not associated with the HRQOL after adjusting for other variables. These results were different from priors studies, which indicated that elder groups, females, separate/divorce/widow people were more likely to have lower HRQOL than others [14, 16, 18, 21, 22, 24, 25]. Several reasons might be used to explain this phenonmenon. Statistically, in the multivariate analysis, we included factors that may covariate with age and gender such as number of health issues and having acute/chronic diseases, adjusting the associations between HQOL and those socio-demographic factors to be insignificant. Otherwise, the insignificant correlation between HRQOL and genders implies that males and females in Vietnam are equal at least on the HRQOL, which is perhaps a result of substantial efforts to improve the gender equality in Vietnam, particularly in health care access and utilization [54]. This is similar to the result in Sri Lanka [5] and Sweden [45]. Likewise, after controlling potential confounders, the likelihood of having better HRQOL is equal to all age groups. It can be due to the facts that in modern life, younger people may be increasingly exposed to diverse harmful factors such as stress, risk behaviors (smoking, alcohol use, opiate drug use or violence), negative interpersonal influences or isolation [31, 32, 55, 56]. Moreover, more and more adolescents and young adults suffer from some diseases such as overweight/obesity and psychiatric disorders, which are rarely found in the elderly populations [57]. In the meantime, the older people, especially in urban areas, promote their independence/autonomy through increasing their social roles; engage in their social activities and have good physical and mental health due to the high quality of health care [58–60]. As a result, elder people can maintain their high HRQOL as the younger ones [58–60].

In this study, people with higher income and being employed had a higher likelihood to get better HRQOL, which was consistent with previous studies [14, 16, 18, 21, 22, 24, 25]. It could be explained that people having jobs and high income were more satisfied with their lives and had higher chance to access health services. However, these findings also suggested the economic disparities in having good HRQOL, which might be more pressing in the future as the Vietnam Ministry of Health has a plan to significantly increase the fee of health services [61]. Moreover, respondents who were well-educated and urban residences were more likely to have a lower HRQOL, which persisted the prior findings in other countries [5, 20, 21]. Several studies revealed that people with higher education and living in urban are more likely to suffer depression/anxiety, which perhaps reduced their HRQOL [62–65]. Additionally, the multivariate analysis affirmed that people having health problems and using health services had a clinically significant reduction in HRQOL compared to their counterparts [26, 34].

This study implies several implications. First, the findings from this study could be used as reference figures, which can enable decision makers to identify the health care needs and burden of diseases; and monitor the effectiveness of policy alterations and provide future investments on health care. Clinicians can use these data to compare the health status of patients with specific conditions to the people with similar socio-economic characteristics [21, 22]. Likewise, the current findings can be used to compare health status among countries. Health economists can also use the data for comparing the difference of HRQOL between the general population and specific patient groups, which could help to calculate quality-adjusted life years in their economic evaluations in Vietnamese settings [40]. Second, offering employment opportunities for retired, unemployed or housework people; providing care promtply for people with illness and raising awareness of elder people about their social roles and promoting their engagement in social activities; and implementing educational interventions to change risk behaviors among young people are several approaches to enhance the HRQOL of the Vietnamese population. Notably, these interventions should not be distinguished between males and females because they are equally vulnerable to have low HRQOL. Finally, further studies to elicit the Vietnamese preference weights for EQ-5D utility index are needed to estimate the HRQOL of the Vietnamese population accurately.

The strength of this study is a large sample size to describe the health status of the general Vietnamese population. However, there are several limitations in our study. First, we only conducted this study in Hanoi, Vietnam; thus, the result may not represent the health status of the population in different settings. In addition, sick individuals may not be recruited into this study due to hospitalization or staying at home. Second, we used the cross-walk value set of Thailand population to derive the utility score of our sample instead of Vietnamese value set. Finally, the cross-sectional design used in this study may constrain the causal relations between HRQOL and other covariates.

## Conclusion

The study informs the first evidence on the HRQOL of general Vietnamese population by using a well-validated instrument namely EQ-5D-5 L. The findings highlight that most of the respondents reported excellent health, and the major health problems were in usual activities and depression/ anxiety. Lower HRQOL were related to unemployed, lower income, higher education, living in urban areas, having acute and chronic diseases and using health service. Further studies should be elucidated to develop value set of Vietnamese population and apply this set to measure population norms.

## Abbreviation

ANOVA: Analysis of variance
EQ-5D-5L: EuroQol – 5 dimensions – 5 levels
GLM: Generalized linear model
HIV: Human Immunodeficiency Virus
HRQOL: Health-related quality of life
MCID: Minimal clinical importance difference
SD: Standard deviation
SEM: Standard error of measurement
VAS: Visual analogue scale
